# Platelet-activating factor acetylhydrolase in primary antiphospholipid syndrome

**DOI:** 10.1007/s13317-018-0103-3

**Published:** 2018-02-10

**Authors:** Paul R. J. Ames, Luis L. Lopez, Mira Merashli, Eiji Matsuura

**Affiliations:** 1 CEDOC, NOVA Medical School, Faculdade de Ciências Médicas, Universidade NOVA de Lisboa, Lisbon, Portugal; 2 Corgenix, Inc. Medical Department, Broomfield, CO USA; 3 Department of Rheumatology, American University of Beirut, Beirut, Lebanon; 4 Department of Cell Chemistry, Okayama University Graduate School of Medicine, Dentistry, and Pharmaceutical Sciences, Neutron Therapy Research Center and Collaborative Research Center for OMIC, Okayama University, Okayama, Japan

Dear Sir,

The interesting article by Fabris et al. shows that individuals screened for antiphospholipid antibodies (aPL) because of a thrombotic or obstetric history exhibit higher platelet-activating factor acetylhydrolase (PAF-AH) in plasma than control blood donors (*p* < 0.0001); amongst the aPL-positive participants, those lupus anticoagulant positive had higher PAF-AH than LA-negative patients (*p* = 0.03) and those positive for IgG anti-beta2 glycoprotein-I antibodies (aβ_2_GPI) presented with higher PAF-AH than patients positive for isolated IgM aβ_2_GPI (*p* = 0.03) [1].

To expand on this topic, we measured PAF-AH in 27 consecutive thrombotic primary antiphospholipid syndrome (PAPS) patients, in 17 thrombotic patients with inherited thrombophilia (IT) and in 23 healthy controls had given written consent for their plasma samples to be stored for research purposes (Table 1). In all participants, we measured IgG anticardiolipin (Cambridge Life Sciences, UK), IgG aβ_2_GPI (Corgenix, Denver, USA), β_2_GPI-oxidised low-density lipoprotein (β_2_GPI-oxLDL) complex and IgG anti-β_2_GPI-oxLDL by previously described immunoassays [2, 3], and PAF-AH by an established method [4]. Lipid profiles were normal in all participants according to measurements done two to three months earlier than the present measurements.

**Table 1:** Demographics and clinical features of the study populations

	CTR	IT	PAPS
Participants (no.)	23	17	27
Female/male	13/7	11/6	18/9
Age (range)	42 (19–55)	40 (30–58)	38 (27–53)
Lupus anticoagulant	0	0	28
IgGaCL (GPL)	3 (1.5–6.0)	3.2 (1.4–6.8)	122 (24–573)
IgGβ_2_GPI (IU)	1 (0.78–3.4)	2 (0.8–4.8)	183 (31–226)
β_2_GPI-oxLDL (IU)	1.6 (1.0–9.0)	1.6 (0.5–6.5)	1.4 (0.8–1.8)
PAF-AH (nmol/ml/min)	46 (22–88)	43 (30–79)	39 (1.8–80)^a^
FVL	0	12	2
PT 20210	1	2	1
PC deficiency	0	3	0
IS	0	2	7
MI	0	0	1
DVT	0	11	14
PE	0	4	5
Smoking	2	2	4
Diabetes	0	0	0
Obesity	0	0	0
Aspirin (75 mg)	0	3	1
Warfarin	0	14	26

All numerical data expressed as median and range. *p* = 0.03 by Kruskal–Wallis ANOVA

*CTR* controls, *IT* inherited thrombophilia, *PAPS* primary antiphospholipid syndrome, *FVL* factor V Leiden, *PT* prothrombin 20210, *PC* protein C, *IS* ischaemic stroke, *MI* myocardial infarction, *DVT* deep vein thrombosis, *PE* pulmonary embolism

^a^ *p* = 0.03

Table 1 shows the results: IgG aPL were elevated by definition in the PAPS group but median PAF-AH was lower in PAPS compared to the other groups (*p* = 0.03); PAF-AH correlated (Spearman rank) positively to β_2_GPI-oxLDL in the CTR (*r* = 0.49, *p* = 0.01) and in the IT (*r* = 0.56, *p* = 0.02) groups but negatively in the PAPS group (*r* = − 0.4, *p* = 0.03). In the latter group, free radical over-generation [5] may inhibit PAF-AH activity [6] perpetuating the effect of PAF that adds to the agonists favouring platelet activation alongside isoprostane [5], thromboxane [7] and thrombin [8]. Our data on low PAF-AH in established PAPS contrast with those of Fabris et al. [1] who do not provide the aPL titres of their screened population and fail to divide participants according to the vascular or obstetric manifestations of APS limiting the interpretation of their data. Our PAPS patients with arterial thrombosis showed a slightly lower PAF-AH than patients with venous thrombosis (33 ± 36 vs 38 ± 61 nmol/ml/min, non significant). In keeping with Fabris [1], we agree that larger studies with clearly defined subsets of patients are required to have a clearer picture on the thrombotic and/or atherogenic role of PAF-AH [9] in PAPS.
